# Vitamin D association with coagulation factors in polycystic ovary syndrome is dependent upon body mass index

**DOI:** 10.1186/s12967-021-02897-0

**Published:** 2021-06-02

**Authors:** Abu Saleh Md Moin, Thozhukat Sathyapalan, Alexandra E. Butler, Stephen L. Atkin

**Affiliations:** 1 Diabetes Research Center (DRC), Qatar Biomedical Research Institute (QBRI), Hamad Bin Khalifa University (HBKU), Qatar Foundation (QF), PO Box 34110, Doha, Qatar; 2 Academic Endocrinology, Diabetes and Metabolism, Hull York Medical School, Hull, UK; 3 Royal College of Surgeons in Ireland Bahrain, Adliya, Kingdom of Bahrain

**Keywords:** Polycystic ovary syndrome, Vitamin D, Obesity, Fibrinogen, Coagulation

## To the Editor:

Polycystic ovary syndrome (PCOS) is associated with metabolic consequences including obesity and insulin resistance that are related to the excess prevalence of type 2 diabetes, hypertension, and cardiovascular diseases in later life [1]. It is reported that PCOS subjects show marked platelet dysfunction [2] and decreased plasma fibrinolytic activity, resulting in a prothrombotic state [3]. In addition, coagulation variables such as thrombin-activatable fibrinolysis inhibitor, plasminogen activator inhibitor-1 (PAI-1), D-dimer, Antithrombin and thrombomodulin have been reported to be elevated in PCOS compared to control subjects [4], and the functional coagulation tests including prothrombin time, thrombin time and fibrin degradation products may be predictive of PCOS [5]. This suggests that PCOS women have a propensity to a hypercoagulable state; therefore, to determine whether this was the case in a cohort of PCOS women, and whether there was an association with vitamin D status, this study was undertaken.

99 PCOS and 68 control Caucasian women “who presented sequentially to the Department of Endocrinology, Hull and East Yorkshire Hospitals NHS Trust were recruited to the local PCOS biobank (ISRCTN70196169) from January 2014 to December 2016. To account for seasonal fluctuation in vitamin D levels, the period of vitamin D sampling in this study was done between March to September of each year that would allow vitamin D targets being achieved through 9 min of sunlight exposure alone in the North of England [6]. The Newcastle & North Tyneside Ethics committee approved this study; all patients gave written informed consent. PCOS diagnosis was based on all three Rotterdam consensus diagnostic criteria [7] and all had a liver ultrasound to exclude non-alcoholic fatty liver disease [8].” None of the women were taking vitamin D supplements at the time of enrolment, nor had they taken any in the 6 months prior to enrolment in the study which was one of the exclusion criteria.

Blood samples were collected and were measured in the Chemistry Laboratory, Hull Royal Infirmary, UK as previously described [8]. Insulin, C reactive protein (CRP) and sex hormone binding globulin (SHBG) were measured by an immunometric assay with fluorescence detection on the DPC Immulite 2000 analyzer using the manufacturer’s recommended protocol [8]. Testosterone was measured by isotope dilution liquid chromatography-tandem mass spectrometry (Waters Corporation, Manchester, UK) as previously described [8]. “The free androgen index (FAI) was calculated as the total testosterone × 100/SHBG. The insulin resistance was calculated using the HOMA method [HOMA-IR = (insulin × glucose)/22.5]. Serum vitamin D levels and testosterone were quantified using isotope-dilution liquid chromatography tandem mass spectrometry (LC–MS/MS)” [8]. Vitamin D sufficiency was defined as > 70 ng/ml, insufficiency as 50–69 ng/ml and deficiency as < 50 ng/ml [8].

Circulating levels of coagulation-related proteins were determined by Slow Off-rate Modified Aptamer (SOMA)-scan plasma protein measurement as previously described [9]. “The SOMAscan assay used to quantify proteins was performed on an in-house Tecan Freedom EVO liquid handling system (Tecan Group, Maennedorf, Switzerland) utilizing buffers and SOMAmers from the SOMAscan HTS Assay 1.3 K plasma kit (SomaLogic, Boulder, CO) according to manufacturer’s instructions and as described previously [9]. Initial Relative Fluorescent Units (RFUs) were obtained from microarray intensity images using the Agilent Feature Extraction Software (Agilent, Santa Clara, CA). Raw RFUs were normalized and calibrated using the software pipeline provided by SomaLogic. Statistical analyses were performed on log_2_ RFU values using R version 3.5.2 (R Foundation for Statistical Computing, Vienna, Austria) including base R package. Data handling and differential protein expression were analyzed using the autonomics and limma and P values were corrected using the Benjamini–Hochberg method.”

Data trends were visually evaluated for each parameter and non-parametric tests were applied on data that violated the assumptions of normality when tested using the Kolmogorov–Smirnov Test. Comparison between groups was performed using Student’s t-test. A p-value of < 0.05 was considered statistically significant. Statistics were performed using Graphpad Prism 8.0 (San Diego, CA, USA).

No power analysis could be performed for this study because there is no data available relating the effect of vitamin D upon coagulation proteins in PCOS.

The PCOS women were older (29.8 ± 0.9 vs. 27.5 ± 0.6 years (± SEM), PCOS vs. control, p = 0.03) and had a higher BMI (p < 0.001), weight (p < 0.0001), waist and hip circumference (p < 0.0001), systolic (p < 0.001) and diastolic (p = 0.03) blood pressure. Biochemically, the PCOS women had elevated anti-mullerian hormone (AMH) (p < 0.0001), triglycerides (p = 0.003), CRP (p < 0.0001), testosterone (p = 0.001), androstenedione (p = 0.003), free androgen index (p < 0.0001), insulin (p < 0.0001) white cell count (p < 0.0001) and platelets (p = 0.01). Vitamin D was significantly lower in the PCOS group (p < 0.0001) and correlated negatively with BMI in PCOS (r = 0.28, p = 0.0046).

Pro-coagulation proteins elevated in PCOS are shown in Table 1 and include higher levels of circulating fibrinogen (p = 0.003), D-Dimer (p < 0.0001), coagulation factor Xa (p = 0.043), integrin alpha1:beta1 (p = 0.017), complement factor 1 (p < 0.0001), p-selectin (p = 0.02) plasminogen activator inhibitor 1 (PAI-1; p < 0.0001), plasma kallikrein (p = 0.0009), fibrinogen gamma chain (p < 0.0001), fibronectin (p = 0.013) and its fragments, fragment 3 (p = 0.03) and 4 (p = 0.004). For anticoagulation proteins, in PCOS, antithrombin was lower (p = 0.002), while PECAM1 (CD31; p = 0.03), tissue type plasminogen activator (p < 0.0001), protein S (p = 0.0008) and heparin cofactor II (p = 0.0014) were higher. Vitamin D correlated negatively with PAI-1 in PCOS (r = 0.24, p = 0.016).

**Table 1:** Coagulation-related proteins in PCOS and control women

	Control (n = 68)	PCOS (n = 99)	p value
Procoagulant			
Fibrinogen	169,317 (2913)	181,362 (2602	**0.003**
D-dimer	12,770 (196)	14,311 (208)	**< 0.0001**
Coagulation factor Xa	5536 (103)	5811 (87)	**0.043**
von Willebrand Factor	13,389 (680)	23,629 (4449)	0.0598
Coagulation factor XI	1801 (33)	1830 (31)	0.54
Prothrombin	164,626 (2344)	159,685 (1588)	0.07
Integrin alpha1:beta1	558 (32)	691 (40)	**0.017**
Complement factor I	41,949 (833)	47,885 (762)	**< 0.0001**
Coagulation factor VII	569 (27)	581 (11)	0.65
P-selectin	12,776 (545)	14,259 (372)	**0.02**
Plasminogen activator inhibitor 1	1495 (134)	2521 (185)	**< 0.0001**
Alpha-2 antiplasmin	1979 (30)	1950 (17)	0.37
Plasma kallikrein	22,653 (517)	24,954 (435)	**0.0009**
Fibrinogen gamma chain	57,240 (1007)	65,382 (1146)	**< 0.0001**
Fibronectin fragment 3	3287 (112)	4702 (540)	**0.03**
Fibronectin fragment 4	68,907 (1699)	84,082 (4181)	**0.004**
Fibronectin	16,170 (808)	27,686 (3759)	**0.013**
Tissue factor	2051 (551)	1872 (362)	0.78
Anticoagulant			
Antithrombin	125,181 (1849)	118,218 (1354)	**0.002**
PECAM1	795 (9)	834 (13)	**0.03**
Tissue type plasminogen activator	469 (22)	627 (21)	**< 0.0001**
Plasminogen	4559 (78)	4547 (80)	0.92
Plasmin	636 (33)	660 (27)	0.56
Protein S	3971 (71)	4385 (88)	**0.0008**
Heparin cofactor II	3824 (77)	4197 (79)	**0.0014**

*PECAM1* platelet endothelial cell adhesion molecule 1

Bolded p-values indicate significance at level of p < 0.05

**Fig. 1:**
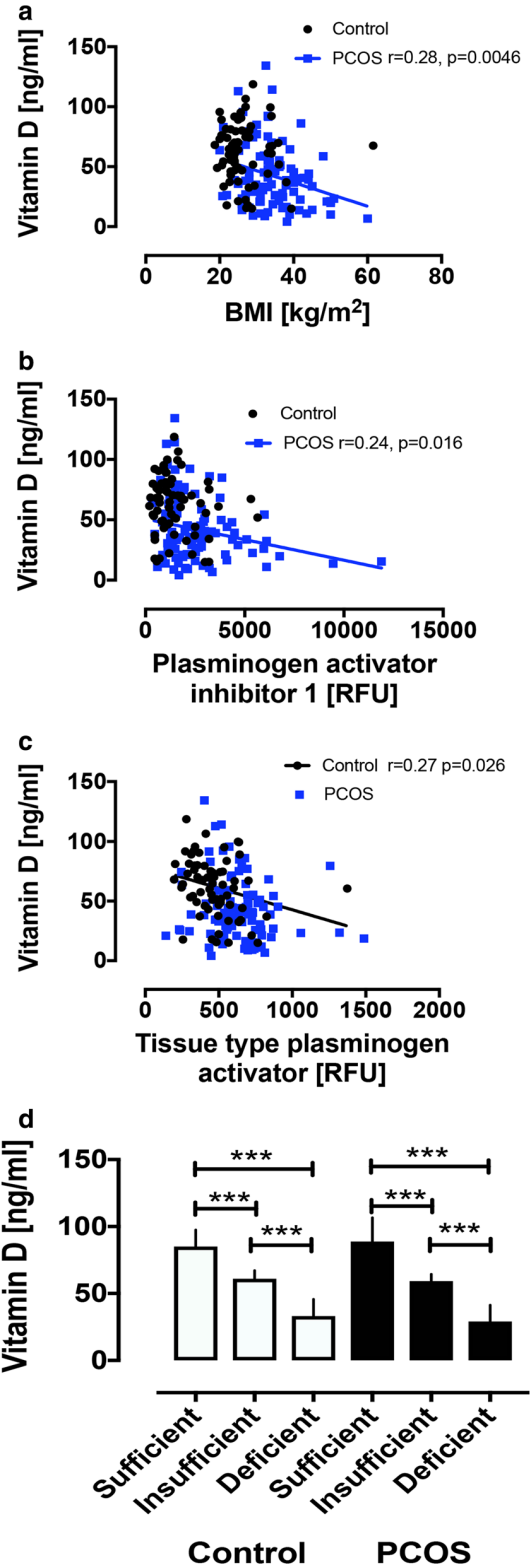
Correlation of vitamin D with BMI (**A**), plasminogen activator inhibitor 1 (**B**) and tissue type plasminogen activator (**C**). Vitamin D concentrations in plasma in PCOS and control women stratified according to sufficient, insufficient and deficient (**D**). ***p < 0.0001

Significant correlations of vitamin D with BMI and coagulation-related proteins are shown in Fig. 1A–C. In the PCOS women, vitamin D correlated negatively with BMI in PCOS, and negatively with plasminogen activator inhibitor 1 (r = 0.24, p = 0.016), while in the control women there was a negative correlation of vitamin D with tissue type plasminogen activator (r = 0.27, p = 0.026). There was no correlation of vitamin D with BMI in control women.

The PCOS and control women were then stratified according to vitamin D status: sufficient, insufficient and deficient. Of the 99 PCOS women, 16 (16%) were sufficient, 11 (11%) were insufficient and 72 (73%) were deficient. Of the 68 control women, 26 (38%) were sufficient, 22 (32%) insufficient and 20 (29%) deficient (Fig. 1D). The only proteins that differed between vitamin D subsets were in controls: tissue plasminogen activator (p = 0.003, sufficient vs. deficient) and integrin alpha1:beta1 complex (p = 0.009, sufficient vs. insufficient) (data not shown). When BMI, inflammation and insulin resistance were accounted for, there was no difference in either the procoagulant or anticoagulant proteins between controls and PCOS in vitamin D deficiency.

These data show that within group analysis in PCOS patients between vitamin D deficient and sufficient patients revealed no alteration in the coagulation or anticoagulation proteins, suggesting that the mechanism of vitamin D deficiency on thrombosis is not through enhanced coagulopathy. In the control patients, anticoagulant tissue plasminogen activator increased (sufficient vs. deficient) and integrin alpha1:beta1 complex (sufficient vs. insufficient) increased. 1,25 Dihydroxyvitamin D has been shown to increase expression of tissue plasminogen activator [10], but the 1,25 dihydroxyvitamin D levels were not measured in this study. No association between vitamin D and integrin alpha1:beta complex has been described before; however, given the large number of comparisons, then a type 1 error cannot be excluded. The data in the controls would be in accord with the studies showing that vitamin D has effects on both the thrombogenic and anti-thrombogenic variables of the coagulation system and has been associated with pulmonary embolus and deep vein thrombosis [11] and that vitamin D supplementation may be protective.

These data are in accord with others in showing that women with PCOS are more vitamin D deficient than those without [8], with 67–85% having serum concentrations of 25-hydroxyvitamin D (25(OH)D) < 20 ng/ml, and levels have been reported to correlate with obesity [8]. These results are also in accord with others who have reported changes in coagulation proteins in PCOS [4], and, here, we show that the changes in the proteins could be accounted for by BMI. Therefore, given the correlation of the coagulation and anticoagulant factors with BMI and the relationship of increased BMI with vitamin D deficiency [12], vitamin D deficiency is likely to be an epiphenomenon to BMI and a marker of BMI in PCOS, rather than having a direct link to enhanced coagulation per se. However, direct effects of hypovitaminosis D cannot be excluded, as seen with the enhanced phosphoprotein enriched in diabetes gene product (PED/PEA-15), independent of obesity in vitamin D deficient PCOS patients that may lead to further downstream ovarian effects [13].

Limitations of this study include that it was a cross-sectional study and that only vitamin D was measured, not the active 1,25 dihydroxyvitamin D. In addition, only the proteins involved in the coagulation pathways were measured and no functional assays were undertaken in this study.

In conclusion, obese subjects with PCOS show a hypercoagulable state and vitamin D deficiency compared to controls; however, there was no difference in the coagulation variables between vitamin D sufficient versus deficient PCOS subjects and BMI accounted for changes in the coagulation proteins, suggesting that BMI rather than vitamin D deficiency may lead to the hypercoagulable state in PCOS.

## Data Availability

All the data for this study will be made available upon reasonable request to the corresponding author.
